# Newer Root Canal Irrigants in Horizon: A Review

**DOI:** 10.1155/2011/851359

**Published:** 2011-11-30

**Authors:** Sushma Jaju, Prashant P. Jaju

**Affiliations:** ^1^Dentocare Multispeciality Dental Clinic, 20 Pratik Arcade near Dena Bank, Bytco Point Nashik Road, Nashik, Maharashtra 422101, India; ^2^Department of Oral Medicine, Diagnosis and Radiology, MGV's KBH Dental College & Hospital, Nashik, Maharashtra, India

## Abstract

Sodium hypochloride is the most commonly used endodontic irrigant, despite limitations. None of the presently available root canal irrigants satisfy the requirements of ideal root canal irrigant. Newer root canal irrigants are studied for potential replacement of sodium hypochloride. This article reviews the potential irrigants with their advantages and limitations with their future in endodontic irrigation.

## 1. Introduction

The effectiveness of endodontic files, rotary instrumentation, irrigating solutions, and chelating agents to clean, shape, and disinfect root canals underpins the success, longevity, and reliability of modern endodontic treatments. The role of microorganisms in the development and perpetuation of pulp and periapical diseases has clearly been demonstrated in animal models and human studies [1–4]. 

Elimination of microorganisms from infected root canals is a complicated task. The chances of a favourable outcome with root canal treatment are significantly higher if infection is eradicated effectively before the root canal system is obturated. However, if microorganisms persist at the time of obturation, or if they penetrate into the canal after obturation, there is a high risk of treatment failure [5, 6].

Numerous measures have been described to reduce the number of microorganisms in the root canal system, including the use of various instrumentation techniques, irrigation regimens, and intracanal medicaments. The use of chemical agents during instrumentation to completely clean all aspects of the root canal system is central to successful endodontic treatment [7]. Irrigation is complementary to instrumentation in facilitating the removal of pulp tissue and/or microorganisms [8]. Irrigation dynamics plays an important role [9, 10]; the effectiveness of irrigation depends on the working mechanism(s) of the irrigant and the ability to bring the irrigant in contact with the microorganisms and tissue debris in the root canal [11, 12]. 

The root canal system is complex and accessory features, such as fins, cul de sacs, and intercanal communications, are colonized by microorganisms once the tooth becomes infected [13, 14]. Self-aggregates of monobacterial morphotypes and coaggregates of different bacterial morphotypes are also found adhering to teeth. The interbacterial spaces are occupied by an amorphous material, spirochetes, and hyphal-like structures that are suggestive of fungi [15, 16]. Costerton et al. used the term “biofilm” to describe this clustering of bacteria [17]. Bacteria within a biofilm have increased resistance to a variety of external hostile influences, such as the host defense responses, antibiotics, antiseptics, and shear forces, compared with isolated bacterial cells [18]. This article reviews recent developments in the identification of new agents to sterilize infected root canal.

## 2. Root Canal Bacterium

Primary root canal infections are polymicrobial, typically dominated by obligatory anaerobic bacteria [19]. The most frequently isolated microorganisms before root canal treatment include Gram-negative anaerobic rods, Gram-positive anaerobic cocci, Gram-positive anaerobic and facultative rods, *Lactobacillus *species, and Gram-positive facultative *Streptococcus *species [20]. The obligate anaerobes are rather easily eradicated during root canal treatment. On the other hand, facultative bacteria such as nonmutans *Streptococci, Enterococci, and Lactobacilli*, once established, are more likely to survive chemomechanical instrumentation and root canal medication [21]. In particular *Enterococcus faecalis *has gained attention in the endodontic literature, as it can frequently be isolated from root canals in cases of failed root canal treatments [22, 23]. In addition, yeasts may also be found in root canals associated with therapy-resistant apical periodontitis [24].

## 3. Root Canal Irrigants

It is generally believed that mechanical enlargement of canals must be accompanied by copious irrigation in order to facilitate maximum removal of microorganisms so that the prepared canal becomes as bacteria-free as possible [25, 26]. Ideally an irrigant should provide a mechanical flushing action, be microbiocidal and dissolve remnants of organic tissues without damaging the periradicular tissues if extruded into the periodontium. In addition, the root canal irrigants should be biocompatible with oral tissues. A large number of substances have been used as root canal irrigants, including acids (citric and phosphoric), chelating agent (ethylene diaminetetraacetic acid EDTA), proteolytic enzymes, alkaline solutions (sodium hypochlorite, sodium hydroxide, urea, and potassium hydroxide), oxidative agents (hydrogen peroxide and Gly-Oxide), local anesthetic solutions, and normal saline [27].

The most widely used endodontic irrigant is 0.5% to 6.0% sodium hypochlorite (NaOCl), because of its bactericidal activity and ability to dissolve vital and necrotic organic tissue [28, 29]. However, NaOCl solutions exert no effects on inorganic components of smear layer. Chelant and acid solutions have been recommended for removing the smear layer from instrumented root canals, including ethylene diaminetetraacetic acid (EDTA), citric acid, and phosphoric acid [30, 31].

**Ideal Requirement of Root Canal Irrigants**

It appears evident that root canal irrigants ideally should [20]

- have a broad antimicrobial spectrum and high efficacy against anaerobic and facultative microorganisms organized in biofilms,
- dissolve necrotic pulp tissue remnants,
- inactivate endotoxin,
- prevent the formation of a smear layer during instrumentation or dissolve the latter once it has formed,
- be systemically nontoxic,
- be non caustic to periodontal tissues,
- be little potential to cause an anaphylactic reaction.

### 3.1. Sodium Hypochlorite

Chlorine is one of the most widely distributed elements on earth. It is not found in a free state in nature, but it exists in combination with sodium, potassium, calcium, and magnesium. In the human body, chlorine compounds are part of the nonspecific immune defense. They are generated by neutrophils via the myeloperoxidase-mediated chlorination of a nitrogenous compound or set of compounds [20]. 

Hypochlorite preparations are sporicidal and virucidal and show far greater tissue dissolving effects on necrotic than on vital tissues. These features prompted the use of aqueous sodium hypochlorite in endodontics as the main irrigant as early as 1920.

There has been much controversy over the concentration of hypochlorite solutions to be used in endodontics. The antibacterial effectiveness and tissue dissolution capacity of aqueous hypochlorite is a function of its concentration, and so is its toxicity [20]. It appears that the majority of American practitioners use “full strength” 5.25% sodium hypochlorite as it is sold in the form of household bleach leading to several adverse reactions like irritation and decrease in flexural strength of dentin. Also decrease in microbiota was also not significantly altered with this high concentration. It must be realized that during irrigation, fresh hypochlorite consistently reaches the canal system, and concentration of the solution may thus not play a decisive role [20]. Unclean areas may be a result of the inability of solutions to physically reach these areas rather than their concentration. Hence, based on the currently available evidence, there is no rationale for using hypochlorite solutions at concentrations over 1% wt/vol. One of the methods to improve the efficacy of sodium hypochlorite was to use heated solution. This improves their immediate tissue-dissolution capacity. Furthermore, heated hypochlorite solutions remove organic debris from dentin shavings more efficiently than unheated counterparts [6]. However, there are no clinical studies available at this point to support the use of heated sodium hypochlorite. Ultrasonic activation of sodium hypochlorite has also been advocated, as this would “accelerate chemical reactions, create cavitational effects, and achieve a superior cleansing action” [20]. However, results obtained with ultrasonically activated hypochlorite versus irrigation alone are contradictory, in terms of both root canal cleanliness and remaining microbiota in the infected root canal system after the cleaning and shaping procedure. it should also be noted that time is a factor that has gained little attention in endodontic studies. Even fast-acting biocides such as sodium hypochlorite require an adequate working time to reach their potential [20]. This should especially be considered in view of the fact that rotary root canal preparation techniques have expedited the shaping process [20]. The optimal time that a hypochlorite irrigant at a given concentration needs to remain in the canal system is an issue yet to be resolved.

### 3.2. EDTA

Although sodium hypochlorite appears to be the most desirable single endodontic irrigant, it cannot dissolve inorganic dentin particles and thus prevent the formation of a smear layer during instrumentation [30]. Demineralizing agents such as ethylenediamine tetraacetic acid (EDTA) and citric acid have therefore been recommended as adjuvants in root canal therapy [30, 31].

These are highly biocompatible and are commonly used in personal care products. Although citric acid appears to be slightly more potent at similar concentration than EDTA, both agents show high efficiency in removing the smear layer [20]. In addition to their cleaning ability, chelators may detach biofilms adhering to root canal walls. An alternating irrigating regimen of NaOCl and EDTA may be more efficient in reducing bacterial loads in root canal systems than NaOCl alone [20]. Antiseptics such as quaternary ammonium compounds (EDTAC) or tetracycline antibiotics (MTAD) have been added to EDTA and citric acid irrigants, respectively, to increase their antimicrobial capacity. The clinical value of this, however, is questionable [32, 33]. Generally speaking, the use of antibiotics instead of biocides such as hypochlorite or chlorhexidine appears unwarranted, as the former were developed for systemic use rather than local wound debridement and have a far narrower spectrum than the latter. Both citric acid and EDTA immediately reduce the available chlorine in solution, rendering the sodium hypochlorite irrigant ineffective on bacteria and necrotic tissue. Hence, citric acid or EDTA should never be mixed with sodium hypochlorite [20].

Calt and Serper demonstrated that 10 mL irrigation with 17% EDTA for 1 minute was effective in removal of smear layer, but a 10-minute application caused excessive peritubular and intertubular dentinal erosion. Increasing contact time and concentration of EDTA from 10% to 17% as well as a pH of 7.5 versus pH 9.0 has been shown to increase dentin demineralization. 

### 3.3. Chlorhexidine

Chlorhexidine was developed in the late 1940s in the research laboratories of Imperial Chemical Industries Ltd. (Macclesfield, England). The original salts were chlorhexidine acetate and hydrochloride, both of which are relatively poorly soluble in water [20]. Hence, they have been replaced by chlorhexidine digluconate. Chlorhexidine is a potent antiseptic, which is widely used for chemical plaque control in the oral cavity. Aqueous solutions of 0.1 to 0.2% are recommended for that purpose, while 2% is the concentration of root canal irrigating solutions usually found in the endodontic literature [20]. It is commonly held that chlorhexidine would be less caustic than sodium hypochlorite. However, that is not necessarily the case [20]. A 2% chlorhexidine solution is irritating to the skin [20]. As with sodium hypochlorite, heating a chlorhexidine irrigant of lesser concentration could increase its local efficacy in the root canal system while keeping the systemic toxicity low. Despite its usefulness as a final irrigant, chlorhexidine cannot be advocated as the main irrigant in standard endodontic cases, because (a) chlorhexidine is unable to dissolve necrotic tissue remnants, and (b) chlorhexidine is less effective on Gram-negative than on Gram-positive bacteria.

## 4. Need for Newer Root Canal Irrigants

All the irrigation solutions at our disposable have their share of limitations and the search for an ideal root canal irrigant continues with the development of newer materials and methods. Newer root canal irrigants in the horizon are as follows:

1. MTAD,
2. tetraclean,
3. electrochemically activated solutions,
4. ozonated water,
5. photon-activated disinfection,
6. herbal irrigants.

The article reviews the advantages and shortcomings of these newer irrigating agents and their potential role in endodontic irrigation in near future.

### 4.1. MTAD

Bio Pure MTAD (Dentsply, Tulsa, OK) is a mixture of a tetracycline isomer, an acetic acid, and Tween 80 detergent (MTAD)—was designed to be used as a final root canal rinse before obturation [32].

Tetracycline has many unique properties of low pH and thus can act as a calcium chelator and cause enamel and root surface demineralization [34]. Its surface demineralization of dentin is comparable to that seen using citric acid [35]. In addition, it has been shown that it is a substantive medication (becomes absorbed and gradually released from tooth structures such as dentin and cementum [33, 35]. Finally, studies have shown that tetracycline significantly enhances healing after surgical periodontal therapy. Manufacturer instructions for using this irrigant were flooding the root canal with 1 mL of the irrigant and soaking for 5 minutes, and the remaining 4 mL is then delivered with continuous irrigation and suction [32]. 

MTAD has some advantages over conventional irrigants and solutions used in root canal treatment. MTAD is effective in removing the smear layer along the whole length of the root canal and in removing organic and inorganic debris and does produce any signs of erosion or physical changes in dentine, whereas a mixture of 5.25% sodium hypochlorite and 17% EDTA does [36–38]. In particular, MTAD mixture is effective against *E. faecalis,* and it is also less cytotoxic than a range of endodontic medicaments, including eugenol, hydrogen peroxide (3%), EDTA, and calcium hydroxide paste [39–42]. 

Torabinejad et al. showed that the effectiveness of the MTAD was enhanced when low concentration of NaOCl is used as an intracanal irrigant before the use of MTAD as a final rinse. MTAD does not seem to significantly change the structure of the dentinal tubules [32].

Newberry et al. determined the antimicrobial effect of MTAD as a final irrigant on eight strains of *Enterococcus faecalis*. After irrigating with 1.3% NaOCl, the root canal and the external surfaces were exposed to MTAD for 5 minutes. Roots or dentin shavings were cultured to determine the growth of *E. faecalis*. The results showed that this treatment regimen was effective in completely eliminating growth in seven of eight strains of *E. faecalis* [43]. Mancini et al. compared the efficacy of Bio- Pure MTAD, 17% EDTA, and 42% citric acid in endodontic smear layer removal and degree of erosion in the apical third of endodontic canals [44]. None of the agents were found to be efficient in the apical one third of the root canal. This finding is in contrast with the results of Torabinejad et al. showing an effective cleaning action with BioPure MTAD in the apical third [32, 36]. The placement of MTAD with a cotton-wrapped barbed broach allows intimate contact of the solution even in the apical region of the canals and improves debridement of the entire root canal wall according to Torabinejad et al. [32].

The effectiveness of sodium hypochlorite, chlorhexidine gluconate, and chlorhexidine acetate on *C. albicans *is documented in the literature [45, 46]. Sen et al. found that EDTA was the most effective irrigant against *C. albicans *using the agar diffusion test [47]. Doxycycline is primarily a bacteriostatic antibiotic and inhibits bacterial protein synthesis by binding to the 30 S bacterial ribosome. Doxycycline is active against a wide range of gram-positive and gram-negative organisms, but it is not active against fungi [48]. As with other antibiotic preparations, use of this drug results in changes in the balance of normal flora and overgrowth of nonsusceptible organisms, including fungi. Citric acid has antibacterial properties but no effect against *C. Albicans* [49]. It follows that antifungal efficacy of MTAD when used in combination with NaOCl may be clinically insignificant. To further improve antisepsis, an additional rinse with chlorhexidine after irrigation with NaOCl may be of benefit [50]. 

Ruff et al. showed that 6% NaOCl and 2% chlorohexidine were equally effective and statistically significantly superior to BioPure MTAD and 17% EDTA in antifungal activity [51]. Clegg et al. questioned the ability of MTAD to remove or disrupt bacterial biofilms in root canals [29, 42]. MTAD is less cytotoxic than eugenol, 3% hydrogen peroxide, calcium hydroxide paste, 5.25% NaOCl, Peridex, and EDTA and more cytotoxic than 2.63%, 1.31%, and 0.66% NaOCl.

MTAD can be a useful irrigant due to its antimicrobial property, less cytotoxic, but its effectiveness against fungi and value in the apical one third need to be assessed further.

### 4.2. Tetraclean

Tetraclean (Ogna Laboratori Farmaceutici, Muggiò (Mi), Italy), like MTAD, is a mixture of an antibiotic, an acid, and a detergent. However, the concentration of the antibiotic, doxycycline (50 mg/mL), and the type of detergent (polypropylene glycol) differ from those of MTAD [52].

Giardino et al. compared the surface tension of 17% EDTA, Cetrexidin, Smear Clear, 5.25% NaOCl, MTAD and Tetraclean [52]. The NaOCl and EDTA had the highest surface tension, whereas Cetrexedin and Tetraclean had the lowest values.

In another study, they compared the antimicrobial efficacy of 5.25% NaOCl, MTAD, and Tetraclean against an *E. faecalis *biofilm generated on cellulose nitrate membrane filters.

Only the NaOCl could disaggregate and remove the biofilm at every time interval tested although treatment with Tetraclean caused a high degree of biofilm disaggregation at each time interval when compared with MTAD [53].

### 4.3. Electrochemically Activated Solutions

Electrochemically Activated (ECA) solutions are produced from tap water and low-concentrated salt solutions [54–56]. 

The ECA technology represents a new scientific paradigm developed by Russian scientists at the All-Russian Institute for Medical Engineering (Moscow, Russia, CIS). Principle of ECA is transferring liquids into a metastable state via an electrochemical unipolar (anode or cathode) action through the use of an element/reactor (“Flow-through Electrolytic Module” or FEM). The FEM consists of an anode, a solid titanium cylinder with a special coating that fits coaxially inside the cathode, a hollow cylinder also made from titanium with another special coating. A ceramic membrane separates the electrodes. The FEM is capable of producing types of solutions that have bactericidal and sporicidal activity; yet they are odourless, safe to human tissue, and essentially noncorrosive for most metal surfaces [54].

Electrochemical treatment in the anode and cathode chambers results in the synthesis of two types of solutions: that produced in the anode chamber is termed an Anolyte, and that produced in the cathode chamber is Catholyte. Anolyte solutions containing a mixture of oxidizing substances demonstrate pronounced microbiocidal effectiveness against bacteria, viruses, fungi, and protozoa [54, 57].

 Anolyte solution has been termed Superoxidized Water or Oxidative Potential Water [58, 59]. Depending on the type ECA device that incorporated the FEM elements the pH of anolyte varies; it may be acidic (anolyte), neutral (anolyte neutral), or alkaline (anolyte neutral cathodic); acidic anolyte was used initially but in recent years the neutral and alkaline solutions have been recommended for clinical application. Under clean conditions, freshly generated superoxidized solution was found to be highly active against all these microorganisms giving a 99.999% or greater reduction in two minutes or less. That allowed investigators to treat it as a potent microbiocidal agent [58, 60].

 It is nontoxic when being in contact with vital biological tissues [61, 62]. 

Clinical applications of anolyte and catholyte were reported to be effective [63]. ECA solutions demonstrated more pronounced clinical effect and were associated with fewer incidences of allergic reactions compared to other antibacterial irrigants tested [63]. Cleaning efficiency and safety for surfaces of dental instruments and equipment has been demonstrated in a number of studies.

The experience of oxidative potential water application for irrigation of root canals has been reported [59]. However, Haga and coworkers studied the effect of acidic anolyte solutions. The anolyte neutral cathodic solution (ANC) provides an increased antiseptic effect and an enhanced cleaning ability at lower concentrations of active chlorine compared to the acidic anolyte and anolyte neutral solutions because of its higher concentration of peroxides. Both electrolyzed neutral water and oxidative potential water are claimed to be harmless to humans and are probably similar to ECA water [33, 37, 54].

The quality of debridement was better in the coronal and middle parts of canal walls where only scattered debris was noted in contrast to the apical part that contained numerous debris. This observation confirms the previously published results [64].

According to Solovyeva and Dummer NaOCl and ECA solutions left a thinner smear layer with a smoother and more even surface [54]. The texture of the canal surfaces treated with ECA solutions was relatively uniform in the various regions of the root canal and did not seem to be influenced by the method of instrumentation, that is, manually or mechanical. Irrigation with NaOCl or ECA solutions enhanced the opening of dentine tubules. It is important to note that irrigation with NaOCL resulted in open tubules predominantly in the coronal and middle thirds of root canals with no signs of tubule orifices were revealed in the apical third of canals. Irrigating with anolyte neutral cathodic as well as with alternate ANC and catholyte resulted in more numerous open dentine tubules in the apical as well as in the coronal regions. Solovyeva and Dummer studied the cleaning effectiveness of root canal irrigation with ECA solution and found that it was similar to NaOCl in debris removal but was more effective than NaOCl in smear layer removal [54].

ECA is showing promising results due to ease of removal of debris and smear layer, nontoxic and efficient in apical one third of canal. It has a potential to be an efficient root canal irrigant. 

### 4.4. Ozonated Water

Ozone is a chemical compound consisting of three oxygen atoms (O_3_–triatomic oxygen), a higher energetic form than normal atmospheric oxygen (O_2_). Thus, the molecules of these two forms are different in structure. Ozone is produced naturally by the following natural methods.

- The first is from electrical discharges following thunderstorms. Ozone is created when an oxygen molecule receives an electrical discharge breaking it into two oxygen atoms. The individual atoms combine with another oxygen molecule to form an O_3_ molecule.
- The second from ultraviolet rays emitted from the sun which plays the role of electrical discharge over oxygen present in the stratosphere, thus, creating the ozone layer which absorbs most of the ultraviolet radiation emitted by the sun.

Ozone is a very powerful bactericide that can kill microorganisms effectively. It is an unstable gas, capable of oxidizing any biological entity. It was reported that ozone at low concentration, 0.1 ppm, is sufficient to inactivate bacterial cells including their spores [65]. It is present naturally in air and can be easily produced by ozone generator. When introduced in water, ozone dissolves rapidly and dissociates rather quickly. The concentration of ozone in ozonated water can be measured using a dissolved ozone meter. Although ozonated water is a powerful antimicrobial agent against bacteria, fungi, protozoa, and viruses, less attention has been paid to the antibacterial activity of ozonated water in bacterial biofilm and hence in root canal infection [66, 67].

Cavitation is the formation of vapor-containing bubbles inside a fluid causing formation of pressure waves/shockwaves characterized by rapid changes in pressure and high amplitude [68]. A forced collapse of bubbles causes implosions that impact on surfaces, causing shear forces, surface deformation, and removal of surface material [69]. In the root canal environment, such shockwaves could potentially disrupt bacterial biofilms, rupture bacterial cell walls, and remove smear layer and debris. Shockwave generation can also enhance the breakdown of agents such as hydrogen peroxide and ozone dissolved in water and thereby enhance their disinfecting and debriding actions [70, 71].

Nagayoshi et al. found that killing ability of ozonated water and 2.5% of sodium hypochlorite was almost comparable when the specimen was irrigated with sonication [72]. 

Study by Hems et al. however found that NaOCI was superior to ozonated water in killing E. faecalis in broth culture and in biofilm [73]. Ibrahim and Abdullah studied that 1.31% NaOCI might allow passage of oxidation of ozonated water, thus increasing their antibacterial effect compared to 1.31% NaOCI or ozonated water alone [67].

Cardoso evaluated the efficiency of ozonated water as an irrigating agent during endodontic treatment in an attempt to eliminate *Candida albicans* and *Enterococcus faecalis *and toneutralize lipopolysacharides (LPSs) inoculated in root canals [16]. It was possible to see effective antimicrobial action after ten minutes of water ozonization on the microbial suspension. There was no residue found when a second sample was collected seven days later. However, ozonated water was not able to neutralize *E. coli *and LPS inside root canals and the remaining amount of LPS may have biological consequences such as apical periodontitis. Estrela et al. assessed the antimicrobial efficiency of aqueous ozone, gaseous ozone, 2.5% sodium hypoclorite, and 2% chlorexidine in human root canals infected with *Enterococcus faecalis*. None of the solutions tested were found to be effective against the bacterial suspension [74, 75].

There is need for further studies and modifications in ozonated water before it could be used as a root canal irrigant.

### 4.5. Photon-Activated Disinfection

The use of photodynamic therapy (PDT) for the inactivation of microorganisms was first shown by Oscar Raab who reported the lethal effect of acridine hydrochloride on Paramecia caudatum [76]. PDT is based on the concept that nontoxic photosensitizers can be preferentially localized in certain tissues and subsequently activated by light of the appropriate wavelength to generate singlet oxygen and free radicals that are cytotoxic to cells of the target tissue [77]. Methylene blue (MB) is a well-established photosensitizer that has been used in PDT for targeting various gram-positive and gram-negative oral bacteria and was previously used to study the effect of PDT on endodontic disinfection [78–85]. Several studies have shown incomplete destruction of oral biofilms using MB-mediated PDT due to reduced penetration of the photosensitizer [86–89]. Soukos et al. used the combined effect of MB and red light (665 nm) exhibited up to 97% reduction of bacterial viability [79]. The results suggested the potential of PDT to be used as an adjunctive antimicrobial procedure after standard endodontic chemomechanical debridement, but they also demonstrated the importance of further optimization of light dosimetry for bacterial photodestruction in root canals. Along with methylene blue, tolonium chloride has been also used as a photosensitizing agent. It is applied to the infected area and left in situ for a short period. The agent binds to the cellular membrane of bacteria, which will then rupture when activated by a laser source emitting radiation at an appropriate wavelength (e.g., 635 nm radiation emitted by SaveDent; Denfotex Light Systems Ltd., Inverkeithing, United Kingdom). The light is transmitted into the root canals at the tip of a small flexible optical fiber that is attached to a disposable handpiece. The laser emits a maximum of only 100 mW and does not generate sufficient heat to harm adjacent tissues. Furthermore, tolonium chloride dye is biocompatible and does not stain dental tissue. The data quoted by the manufacturer suggest that this PAD system has antimicrobial efficacy [90]. Lethal photosensitization of *Streptococcus intermedius *biofilms in root canals is unable to achieve a total kill rate when a combination of a helium-neon laser and tolonium chloride is used [91].

Leticia et al. investigated the antibacterial effects of photodynamic therapy (PDT) with methylene blue (MB) or toluidine blue (TB) (both at 15 mg/mL) as a supplement to instrumentation/irrigation of root canals experimentally contaminated with *Enterococcus faecalis* [92]. The study revealed that PDT with either MB or TB may not exert a significant supplemental effect to instrumentation/irrigation procedures with regard to intracanal disinfection, until further adjustments in the PDT protocol are modified before clinical use is recommended.

In contrast, irrigation with sodium hypochlorite (3%) eliminated the entire bacterial population. The difference could be because the optical fiber was not properly introduced into the root canals, and so the light could not transmit through the tooth structure. Thus, PAD might not be able to achieve a 100% kill rate in infected root canals that have complex anatomic features and colonized by polymicrobial biofilms of varying properties. 

Pagonis et al. studied the in vitro effects of poly(lacticco- glycolic acid) (PLGA) nanoparticles loaded with the photosensitizer methylene blue (MB) and light against *Enterococcus faecalis* (ATCC 29212) [93]. The study showed that utilization of PLGA nanoparticles encapsulated with photoactive drugs may be a promising adjunct in antimicrobial endodontic treatment.

 PAD can currently be considered a useful adjunct to conventional root canal treatment.

### 4.6. Herbal

Murray et al. evaluated Morinda citrifolia juice in conjunction with EDTA as a possible alternative to NaOCl. Triphala (IMPCOPS Ltd, Chennai, India) is an Indian ayurvedic herbal formulation consisting of dried and powdered fruits of three medicinal plants, Terminalia bellerica, Terminalia chebula, and Emblica officinalis, and green tea polyphenols (GTPs; Essence and Flavours, Mysore, India); the traditional drink of Japan and China is prepared from the young shoots of tea plant Camellia sinensis [94–97]. 

Dimethyl sulfoxide (DMSO) is used as a solvent for Triphala and GTP, although they are readily soluble in water. DMSO is a clean, safe, highly polar, aprotic solvent that helps in bringing out the pure properties of all the components of the herb being dissolved [98, 99].

Herbal alternatives showed promising antibacterial efficacy on 3- and 6-week biofilm along with MTAD and 5% sodium hypochlorite [97].

Although Triphala and green tea polyphenols (GTPs) exhibited similar antibacterial sensitivity on *E. faecalis* planktonic cells, Triphala showed more potency on *E. faecalis* biofilm. This may be attributed to its formulation, which contains three different medicinal plants in equal proportions. In such formulations, different compounds may be of help in enhancing the potency of the active compounds resulting in an additive or synergistic positive effect. According to Prabhakar et al. 5% of sodium hypochlorite exhibited excellent antibacterial activity in both 3-week and 6-week biofilm, whereas Triphala and MTAD showed complete eradication only in 3-week biofilm [97].

 Triphala and GTPs are proven to be safe, containing active constituents that have beneficial physiologic effect apart from its curative property such as antioxidant, anti-inflammatory, and radical scavenging activity and may have an added advantage over the traditional root canal irrigants [100–103].

## 5. Conclusion

The article reviewed the potential new irrigants that could substitute the traditional endodontic irrigants. Available literature and studies demonstrate advantages and limitations of each irrigant under consideration and none of them satisfy the requirements of the ideal root canal irrigant completely. Presently these newer irrigants could be used as an adjunct to NaOCl, with the hunt for the elusive ideal root canal irrigant continues.
